# Preoperative prognostic nutritional index predicts postoperative surgical site infections in gastrointestinal fistula patients undergoing bowel resections

**DOI:** 10.1097/MD.0000000000004084

**Published:** 2016-07-08

**Authors:** Qiongyuan Hu, Gefei Wang, Jianan Ren, Huajian Ren, Guanwei Li, Xiuwen Wu, Guosheng Gu, Ranran Li, Kun Guo, Youming Deng, Yuan Li, Zhiwu Hong, Lei Wu, Jieshou Li

**Affiliations:** a Department of Surgery, Jinling Hospital, Medical School of Southeast University, Nanjing, China; b Medical School of Nanjing University, Nanjing, China.

**Keywords:** GI fistula, prognostic nutritional index, surgical site infections

## Abstract

Recent studies have implied a prognostic value of the prognostic nutritional index (PNI) in postoperative septic complications of elective colorectal surgeries. However, the evaluation of PNI in contaminated surgeries for gastrointestinal (GI) fistula patients is lack of investigation. The purpose of this study was to explore the predictive value of PNI in surgical site infections (SSIs) for GI fistula patients undergoing bowel resections.

A retrospective review of 290 GI patients who underwent intestinal resections between November 2012 and October 2015 was performed. Univariate and multivariate analyses were conducted to identify risk factors for SSIs, and receiver operating characteristic cure was used to quantify the effectiveness of PNI.

SSIs were diagnosed in 99 (34.1%) patients, with incisional infection identified in 54 patients (18.6%), deep incisional infection in 13 (4.5%), and organ/space infection in 32 (11.0%). receiver operating characteristic curve analysis defined a PNI cut-off level of 45 corresponding to postoperative SSIs (area under the curve [AUC] = 0.72, 76% sensitivity, 55% specificity). Furthermore, a multivariate analysis indicated that the PNI < 45 [odd ratio (OR): 2.24, 95% confidence interval (CI): 1.09–4.61, *P* = 0.029] and leukocytosis (OR: 3.70, 95% CI: 1.02–13.42, *P* = 0.046) were independently associated with postoperative SSIs.

Preoperative PNI is a simple and useful marker to predict SSIs in GI fistula patients after enterectomies. Measurement of PNI is therefore recommended in the routine assessment of patients with GI fistula receiving surgical treatment.

## Abstract 2

Supplemental Digital Content is available in the text

## 1 Introduction

Surgical site infections (SSIs) are deemed the third most common hospital-acquired infection, and elective colorectal surgery has the highest incidence of SSIs.^[1]^ Because SSIs have been reported to pose an additional financial burden and increase length of the postoperative hospital stays, delineation of the incidence of SSIs and recognition of their risk factors are extremely important. Gastrointestinal (GI) fistula operations, which are commonly contaminated surgery, have higher risk of SSIs compared with other colorectal interventions. Additionally, intricate medical and surgical conditions for GI fistula patients collectively contribute to the development of an SSI. Therefore, early diagnosis and management of SSIs after GI fistula surgeries deserves our sufficient attention.

Clinically, indices containing various nutritional and inflammatory parameters were used to predict surgical risks and postoperative complications.^[2]^ The prognostic nutritional index (PNI) advocated by Onodera et al,^[3]^ calculated based on the serum albumin concentration and peripheral blood total lymphocyte count (TLC), was the simplest parameter among previously reported prognostic nutrition indices for determining the nutritional and inflammatory status of surgical patients. This formula was an established prognostic parameter for various types of cancer, which has been validated previously to evaluate the risk of postoperative complications and mortality in GI tract surgery.^[4–6]^ A recent study also revealed that a preoperative low PNI correlated with the occurrence of incisional SSIs after bowel resections in patients with Crohn disease.^[7]^

So far no reports have been published regarding association between PNI and SSIs in GI fistula patients undergoing intestinal resections. Thus, the primary objective of this study was to clarify the prognostic value of PNI in patients with GI fistula.

## 2 Materials and methods

### 2.1 Patients

This was a retrospective, observational study carried out in GI Fistula Center of Department of Surgery, Jinling Hospital, China. GI fistula patients who underwent abdominal surgeries from November 2012 to October 2015 were enrolled in this study. The study was approved by the institutional review board of Jinling Hospital. Consent from participants was not required in this retrospective study.

**Figure 1:**
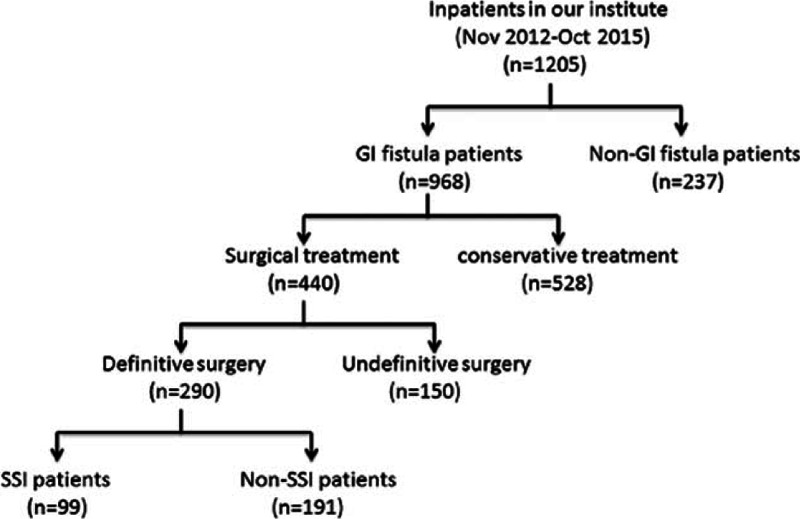
Enrollment flowchart. A total of 968 patients with gastrointestinal fistula (GI) were collected from 1205 inpatients in our institute between November 2012 and October 2015. Among 440 GI patients receiving surgical procedure, 290 received definitive surgery and were divided into surgical site infections (SSI) group (n = 99) and non-SSI (n = 191) groups.

The inclusion criteria of patients were be at least 18 years of age; having an abdominal CT scan or fistulography that confirmed diagnosis of GI fistula, and being scheduled for GI fistula resections. All anastomoses were created using a linear stapler. A total of 290 GI fistula patients were enrolled in the analysis (Fig. 1).

### 2.2 Data collection and laboratory measurements

The following data of enrolled patients collected by the same physician were retrieved from the medical record system of Jinling hospital: demographic characteristics [age, sex, body mass index, history of smoking, and alcohol consumption]; etiopathogenies including malignancy, inflammatory bowel disease, pancreatitis, road traffic accidents, or trauma; surgical approach (open or laparoscopic); intraoperative blood loss; duration of operation; types of colectomies; and American Association of Anesthesiology score, methods of anastomosis, and number of anastomoses.

Blood of GI fistula patients was drawn preoperatively at 5:00 am on the day of surgery. Routine laboratory measurements included Hemoglobin (Hb), white blood cell count, platelet count, C-reactive protein (CRP), procalcitonin (PCT), and serum albumin. According to the WHO, anemia is defined as Hb level less than 12 g/dL for nonpregnant females and less than 13 g/dL for males; leukocytosis was defined as a white blood cell greater than 11  × 10^9^/L and serum albumin less than 3.5 g/dL was considered as hypoalbuminemia. The PNI was estimated according to the following formula: 10 × serum albumin (g/dL) + 0.05 × TLC (/mm^3^).

### 2.3 Preoperative management and outcome measures

When the GI patients were admitted in our institution, we initially corrected or maintained their hydroelectrolytic equilibration, controlled the infections using proper surgical drainage or antibiotics and reinforced patients’ nutritional status using parental nutrition or enteral nutrition. Moreover, a special modified double cannula drainage was used to improve drainage in our intestinal fistula center.^[8]^ The purpose of the preoperative management was to improve patients’ mental, nutritional, and inflammatory conditions. In the end, a definitive operation was subsequently considered to solve the existing fistula.

The major outcome of interest in the present study was incidence of SSIs. All SSIs events were defined on the basis of guidelines issued by National Nosocomial Infection Surveillance system of the Centers for Disease Control and Prevention.^[9]^ SSIs were defined as occurrence of an infection within 30 days after operation, further classified as superficial incisional, deep incisional, and organ space infections. At our institution, SSIs were identified according to at least one of the following criteria: purulent drainage from the surgical site; an organism isolated from a culture of fluid from the surgical site; incisional pain, tenderness, localized swelling, redness, or heat and opening of the wound; or diagnosis of SSI by the surgeon or attending physician.^[7,10]^ In the present study, anastomotic leakage or intra-abdominal abscesses after surgery was considered as an organ/space SSI.

### 2.4 Statistical analysis

Statistical analyses were performed using SPSS 19.0 statistical software (SPSS Inc., Chicago, IL). Potential risk factors of SSIs were assessed by univariate and multivariate analyses. Univariate analyses were performed using the Chi-square test or Fisher's exact test. To better clarify the independent factors of SSIs in GI patients, all variables with a value *P* < 0.25 in the univariate analyses were enrolled into a multivariate logistic regression analysis. Comparisons of PNI and CRP were done by receiver operating characteristic (ROC) and binary logistic regression were also performed in SPSS. The thresholds were developed with an equal emphasis on sensitivity and specificity with the Youden index. The area under curve (AUC) was used to quantify the effectiveness of PNI and CRP levels in predicting postoperative SSIs. *P* < 0.05 was considered to be statistically significant.

## 3 Results

### 3.1 Clinical characteristics of the enrolled patients

The clinical and demographic information from 290 patients with GI fistula who underwent intestinal resections in our institution were summarized in Table 1. Surgical patients with SSIs have longer length of hospital stays than non-SSI group (33 ± 9.6 vs 25 ± 8.8 days, *P* < 0.001) and statistical difference regarding the total cost of hospitalization was found between 2 groups (116 ± 23.8 vs 155 ± 32.7, × 1000, Chinese Yuan). Table 2 showed relationships between the laboratory parameters and SSIs. There were significant intergroup differences in the serum levels of CRP (*P* = 0.012), PCT (*P* = 0.012) and TLC (*P* = 0.019), preoperative leukocytosis (*P* = 0.013), hypoalbuminemia (*P* = 0.001), and levels of PNI (*P* < 0.001). Moreover, the incidence of leukocytosis in SSI group (9.1%) increased more than 4-fold compared with patients in non-SSI group (2.1%). Table 3 showed the comparisons of fistula characteristics between 2 groups and no statistical significance was found. There were 127 GI fistula patients that developed postoperative complications, and more than three-quarters (78%) were SSIs (Supplementary Table S1).

**Table 1:**
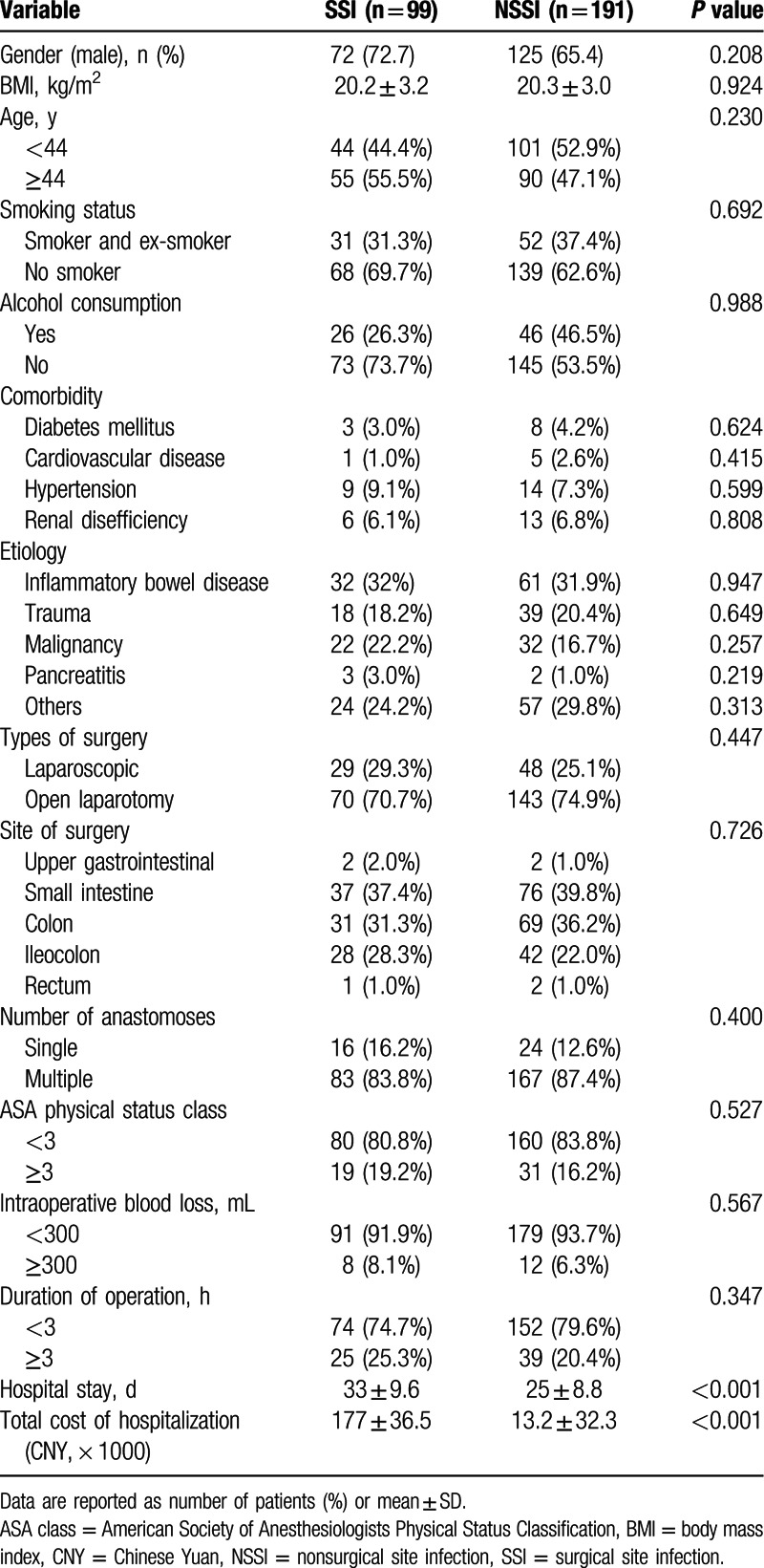
Demographic and clinical characteristics of GI fistula patients.

**Table 2:**
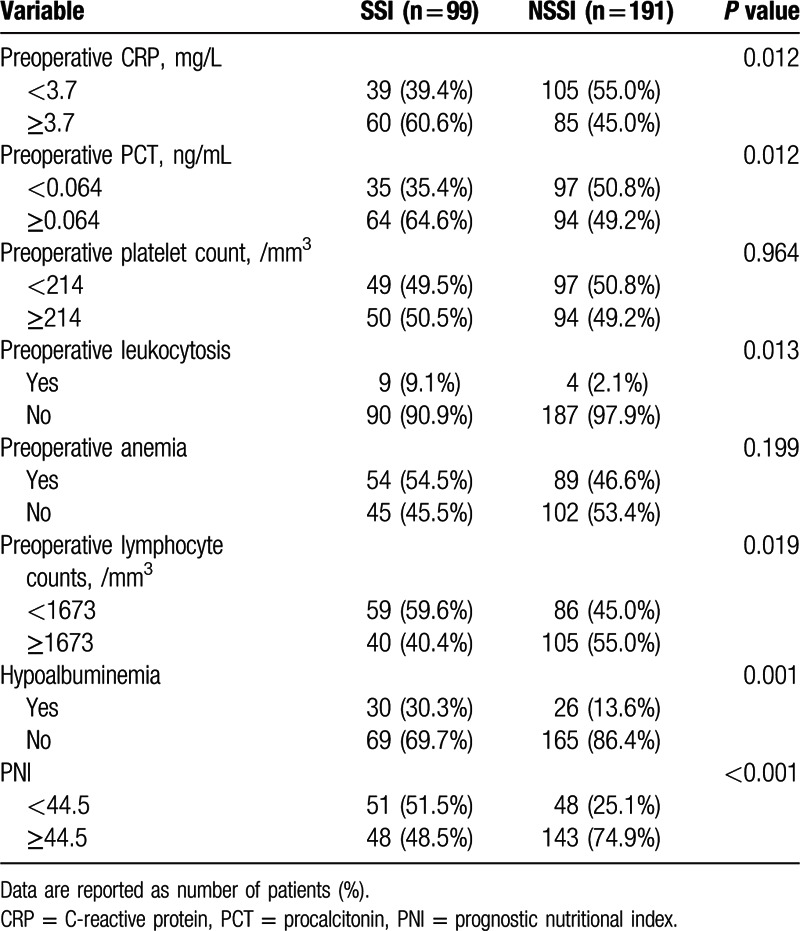
The relationships between the laboratory features and surgical site infections.

**Table 3:**
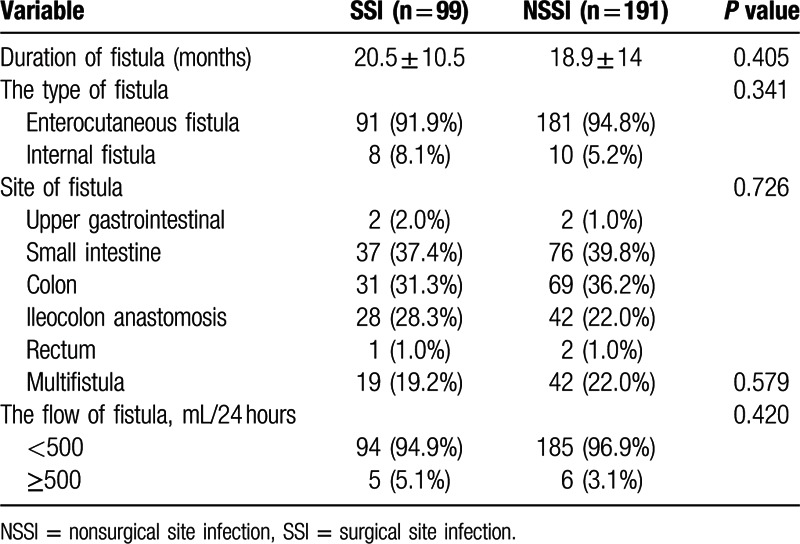
Fistula characteristics.

### 3.2 ROC cure analysis

**Figure 2:**
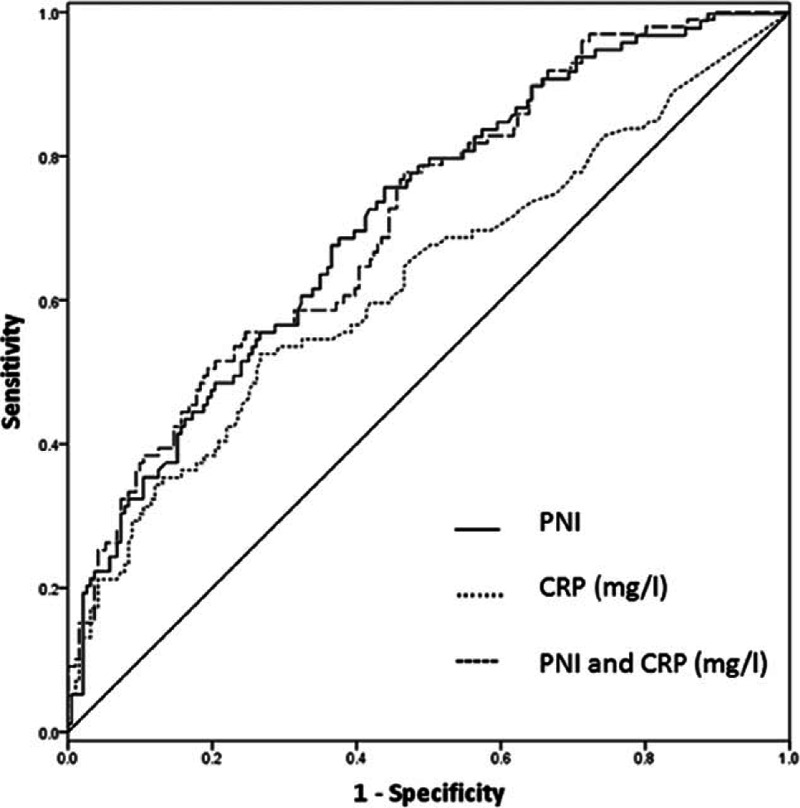
Receiver operating characteristic (ROC) curve analysis for prognostic nutritional index (PNI) and C-reactive protein (CRP) values as risk predictors of postoperative surgical site infections in GI patients underwent enterectomies.

Serum CRP, as an acute phase protein synthesized by liver, was regarded as a key factor of SSIs in patients with colorectal surgeries.^[11]^ In the present study, ROC analysis was performed to evaluate the performance of PNI and CRP levels in discriminating SSIs. The optimal cutoff value of PNI was determined at 45 and the AUC was 0.72 corresponding to the sensitivity and specificity of 76% and 55%, respectively. The AUC of CRP levels was 0.63 with 65% sensitivity and 52% specificity. In order to investigate whether the combination of PNI and CRP in clinical practice improved the prediction of SSIs, we performed binary logistic regression analysis including both parameters. The combination of PNI and CRP was not better than PNI alone to discriminate SSIs (AUC = 0.72, 79% sensitivity and 39% specificity; Fig. 2).

### 3.3 The association of preoperative PNI with postoperative SSIs

The overall incidence of SSIs for GI patients was 34.1 % (99/290). Incisional SSIs were observed in 54 patients (18.6%), deep incisional SSIs in 13 patients (4.5%), and organ/space SSIs in 32 (11.0%) patients (Table 4). Moreover, we found that the frequency of overall, incisional and organ/space SSIs were evidently higher in low PNI group. Nevertheless, no significant correlation was found between deep incisional SSIs and PNI.

**Table 4:**
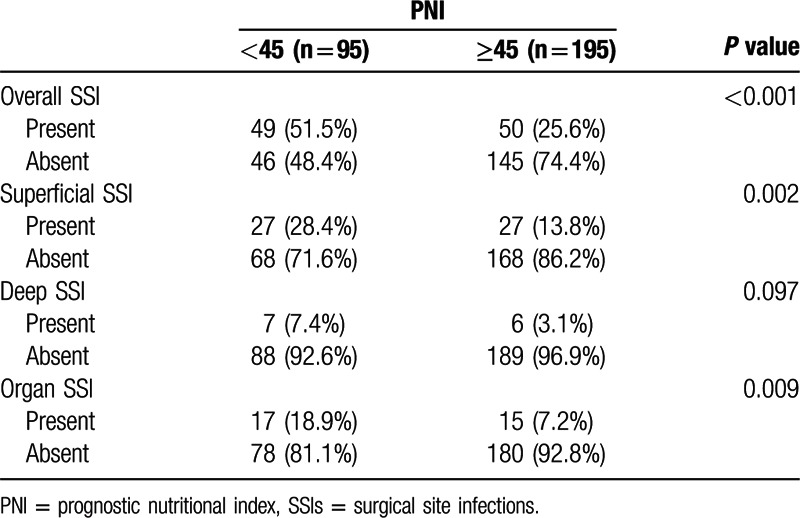
Correlation between SSIs and the PNI.

All variables with *P* < 0.25 in the univariate analyses were selected into a multivariate logistic regression analysis. Multivariate analyses revealed that a preoperative low PNI (*P* = 0.029, odds ratio [OR] = 2.24, 95% confidence interval [CI] = 1.09–4.61) and leukocytosis (*P* = 0.046, OR = 3.70, 95% = 1.02–13.42) were independently associated with postoperative SSIs in GI patients undergoing bowel resections. The preoperative CRP, PCT, TLC, and hypoalbuminemia were not found to be significant factors for the occurrence of SSIs (Table 5).

**Table 5:**
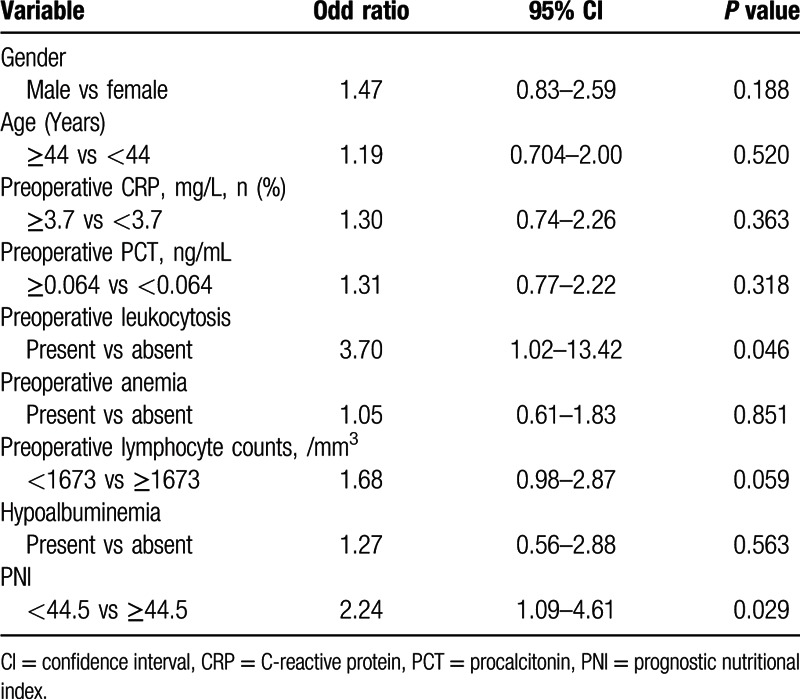
Multivariate analyses according to the correlation between various clinical variables and the incidence of surgical site infections.

## 4 Discussion

In our study, we explored the predictive value of PNI and CRP levels among a considerable GI fistula cohort and found that preoperative PNI performed better than CRP in predicting SSIs after enterectomies. SSIs are one of the most common postoperative complications in patients undergoing colorectal surgery and contribute to increased postoperative morbidity, longer hospital stay, and health care burden.^[12]^ Therefore, preoperative measurements of PNI and leukocytosis may help identify GI fistula patients at high risk of developing SSIs after surgery.

The identification of risk factors for SSIs in patients receiving GI surgery remains debatable. To our knowledge, previously reported potential risks for SSIs included longer operative time, intraoperative blood loss, diabetes, and American Association of Anesthesiology score.^[13]^ However, the association did not exist between these risk factors and SSIs in the present study, which may result from complicated medical and surgical conditions in GI fistula patients. Additionally, preoperative CRP, PCT, TLC, and hypoalbuminemia were found to be significant factors associated with SSIs emerging from univariate analysis. These factors, however, were not identified to be independent factors by the multivariate analyses. Only preoperative PNI and leukocytosis were proved to independently predict the development of postoperative SSIs.

About 13 (4.5%) GI patients presented preoperative leukocytosis in our cohort (Table 1). Leukocytosis, a marker of inflammation and cytokine release, may contribute to medical comorbidities or surgical complexity.^[14]^ Recent studies investigated the prognostic role of leukocytosis in disease progression of some malignancies.^[15]^ Also, preoperative leukocytosis turned out to be a negative prognostic indicator associated with infectious complications of intra-abdominal infections and deep incisional SSIs.^[16]^ In the present study, preoperative leukocytosis was presented to be an independent factor predicting postoperative SSIs. Preoperative leukocytosis in GI patients receiving surgical treatment, based on our results, provided practical guidance to the presence of SSIs. Furthermore, larger-scale and prospective clinical trials are required to verify the clinical utility of leukocytosis.

Patients with GI fistula are often malnourished owing to losing intestinal juice and electrolytes, so performing adequate nutritional assessments in such patients is therefore necessary. Some assessment tools, such as the Malnutrition Universal Screening Tool, the Nutritional Surgical Risk Index, and the Nutritional Risk Scoring 2002, can be utilized to evaluate the nutritional condition.^[17,18]^ These are very practical and cost-effective indices that are widely applied to evaluate the nutritional status. Compared with other parameters, the PNI is a simpler index only calculated by the serum albumin concentration and TLC, which are routine parameters assessed in the clinical setting. Serum albumin level has been regarded as a good index of malnutrition in a wide variety of patient populations,^[7,19]^ and the TLC has been considered as a helpful indicator of systemic inflammatory status.^[3]^ Thus, we believed that the preoperative PNI, which reflected both nutritional and inflammatory status, could be used to predict the development of SSIs in GI patients undergoing intestinal resections. Several studies have revealed that a low PNI was significantly related to the high occurrence of SSIs in patients undergoing GI operation.^[20,21]^ Nozoe et al^[22]^ reported that the mean preoperative PNI in esophageal cancer patients with postoperative complications was significantly lower than that in those without postoperative complications. According to our findings, although PNI was not associated with deep incisional SSIs, PNI < 45 was a significant independent risk factor for overall SSIs using multivariate analysis. Hence, physician should attach great importance to perioperative care for patients with a low PNI value.

There is increasing evidence that the presence of a systematic inflammatory response, as indicated by an elevated circulating CRP concentration, is associated with postoperative complications (including SSIs) in patients with abdominal surgery.^[23,24]^ In this study, we evaluated the diagnostic accuracy of CRP level and PNI in GI fistula cohort and found that PNI (AUC = 0.72) performed better than CRP (AUC = 0.63) in discriminating SSIs. Beyond that, PNI in combination with CRP did not enhance the discriminative power. Preoperative CRP levels, contrary to a previous research,^[25]^ were not an independent risk factor in predicting SSIs. These data suggested that PNI could be considered as a new significant marker to assess the risks in occurrence of SSIs.

This study is subject to limitations: first, this was a retrospective and single-center study, which may also have potential flaws in the accuracy of documentation in the medical records. Second, details concerning operative procedure, such as time of using antibiotics, core body temperature, and degree of contamination were not evaluated in our study. Despite these limitations, to our knowledge, this is the first study to evaluate the prognostic significance of preoperative PNI in GI fistula patients undergoing intestinal resections.

In conclusion, higher PNI in GI patients suggests a favorable prognosis. Considering that GI fistula patients at higher baseline risk for SSIs, preoperative PNI should be included in essential assessment of GI patients receiving enterectomies and patients with a lower PNI should monitored and receive targeted medical care to avoid postoperative SSIs.

## Acknowledgments

Chunyu Jian and Huan Su from affiliated hospital of southeast university greatly helped to make this study a reality when we started our study. I also owe my sincere appreciation to Chunyu Jian and Huan Su, who gave support and energy to me during the difficulty of the study.

## Supplementary Material

Supplemental Digital Content
