# Supplementary material for: Preoperative prognostic nutritional index predicts postoperative surgical site infections in gastrointestinal fistula patients undergoing bowel resections
Source: Medicine (Baltimore). 2016 Jul 8;95(27):e4084. doi: 10.1097/MD.0000000000004084 (PMC5058827; doi:10.1097/MD.0000000000004084)
Supplement: Supplemental Digital Content [file medi-95-e4084-s001.pdf]

Supplementary Table 1. Postoperative Complications

**Postoperative complications**	**Number**
**Surgical site infections, n (%)**	**99(78.0%)**
Incisional infection	54
Deep incisional infection	13
Organ/space infection	32
**Other than surgical site infections, n (%)**	**28(22.0%)**
Inflammatory ileus	9
Bleeding	6
Cardiac problems	2
Urinary tract infection	2
Pneumonia	2
Acute liver injury	1
CRBSI	1
Hydrothorax	1
Cholestasis cholecystitis	1
Deep vein thrombosis	1
Acute kidney injury	1
Phlebitis	1
**Total**	**127**

CRBSI: Catheter Related Blood Stream Infection
